# From the Hospital Bed to the Laptop at Home: Effects of a Blended Self-Regulated Learning Intervention

**DOI:** 10.3390/ijerph16234802

**Published:** 2019-11-29

**Authors:** Raquel Azevedo, Pedro Rosário, Juliana Martins, Daniela Rosendo, Paula Fernández, José Carlos Núñez, Paula Magalhães

**Affiliations:** 1 Department of Applied Psychology, Escola de Psicologia, Universidade do Minho, Campus Gualtar, 4710-052 Braga, Portugal; prosario@psi.uminho.pt (P.R.); julianaoliveiramartins1992@gmail.com (J.M.); danielapatriciamr@gmail.com (D.R.); pmagalhaes@psi.uminho.pt (P.M.); 2 Department of Psychology, Universidad de Oviedo, Plaza Feijoo s/n., 33003 Oviedo, Spain; paula@uniovi.es (P.F.); jcarlosn@uniovi.es (J.C.N.); 3 Facultad de Ciencias Sociales y Humanidades, Universidad Politécnica y Artística de Paraguay, Mayor Sebastián Bullo s/n, Asunción, Paraguay

**Keywords:** hospitalization, school-aged children and adolescents, self-regulated learning, school engagement, blended learning, technology, intervention

## Abstract

Hospitalization poses diverse challenges to school-aged youth well-being and their educational path. Some inpatients, due to the hospitalization duration, frequency or the needed recovery period at home, may struggle when returning to school. To help youth cope with this challenge, several hospitals have been implementing educational interventions tailored to the school-aged children and adolescents needs. Nevertheless, pediatric inpatients with short stays and/or with a recovery period at home usually do not benefit from these interventions. Therefore, the present study implemented a blended intervention (i.e., face-to-face and online) with the aim of training self-regulated learning competences with hospitalized school-aged adolescents with short hospital stays. The intervention was delivered on a weekly basis for eight individual sessions using a story-tool. Results showed the efficacy of the intervention in promoting adolescent’s use of, perceived instrumentality of, and self-efficacy for self-regulated learning strategies. Overall, there was a differentiated impact according to the participants’ age, grade level, grade retention, and engagement in the intervention. These findings support previous research indicating that hospitals can play an important role as educational contexts even for inpatients with short stays. The blended format used to deliver the self-regulation learning (SRL) training also may be an opportunity to extend these interventions from the hospital to the home context.

## 1. Introduction

Being hospitalized, either for extended periods or repeated admissions, poses great challenges to school-aged children’s school experience [1], social well-being, and continued educational opportunities [2]. It might also increase the risk for school failure and disengagement from school [2]. Recognizing this impact, several hospitals have been implementing interventions, e.g., [3,4], to address the pediatric inpatients needs (e.g., health-education and curricular content). However, many of these interventions are not available for all inpatients because hospital stays are short and, in some cases, there is a recovery period at home before returning to school [5,6]. In view of this scenario, technology may play an important role [7] in supporting the educational interventions provided to hospitalized youth [8], specifically those with short hospital stays. The present study describes and evaluates a blended intervention on self-regulated learning for hospitalized school-aged youth. 

### 1.1. Hospitalization: Consequences and Impact in General

Literature on the adverse impact of hospitalization, e.g., [9,10,11], either short or long term, on children and adolescents’ lives is vast. Extant research indicates that hospitalization can be a stressful and traumatic experience for children [12,13,14,15,16,17] and adolescents [18], with extended implications for the family, e.g., [12,19,20]. Caires et al. [21] refer three main stressors in this context: (i) the distancing of the child from their main life contexts; (ii) the considerable changes in their routines; and (iii) the perceived menace regarding their clinical situation and necessary medical procedures. Incontestably, hospitalization constitutes a disruption on the normalcy of the children and adolescents’ everyday lives, placing them at risk of becoming socially isolated from their school community [22], friends, and family [23]. What is more, upon discharge, returning to normalcy may be a challenge [10,24]. Acknowledging the influence that contexts play in child and adolescent development, it becomes crucial to learn the possible consequences that hospitalization may play in their development [20]. A study by Burns-Nader [25] examined children’s anxiety, and feelings of dependency, towards either a doctor’s appointment or a short hospitalization. Results suggested that, despite the short-term duration of the hospitalization, children still reported feeling more anxious and dependent during this event than in a consultation. Amongst the reasons that could explain children’s anxiety regarding hospitalization are contact with strange people, distance from home and family, and the high vulnerability and uncertainty about their health condition in the future [26]. Importantly, the time of admission seems to be the period where patient stress is heightened, which may help explain the anxiety levels exhibited by youth even in short-term hospitalizations [27]. Furthermore, literature indicates that children whose health situation involves short, frequent and/or prolonged hospitalizations, or even recovery periods at home, have fewer opportunities for the stimulation of competences transversal to their daily activities [8,10]. These opportunities for training transversal skills are fundamental for behavioral, emotional, and cognitive self-regulation. This suggests that it is essential to reduce hospitalization time to allow children to return to their normative life contexts [28], and use the hospitalization period, whenever possible, as an opportunity for health recovery and training competences.

### 1.2. Hospitalization: Impact on the Educational Path of the Adolescent

In the U.S., about two-thirds of school-aged children miss school due to illness/accident [29], implying an involuntary withdrawal of school activities that can impact and delay their learning process [30]. In Portugal, it is estimated that school-aged children and adolescents are hospitalized 20,000 days per year [31]. Moreover, in Portugal, in 2017, there were on average 3400 hospitalizations of children between 5 and 9 years old, 7100 of children between 10 and 14, and 9500 of adolescents between 15 and 17. The average length of a hospital stay in Portugal, in 2015, was 3.8 days (5 to 9 years of age), 4.4 days (10 to 14 years of age), and 5 days (15 to 19 years of age) [32]. Furthermore, data from the OECD encompassing all types of internment show that among OECD countries, the average length of hospital stays was approximately 8 days in 2017 [33]. Specifically, Portugal has a general average length of 7.3 days, with Turkey in the lower end with 4.2 days, United States with 5.5 days, and Japan with 16.2 days at the higher end [34].

School represents a significant part of a youth’s life [35]. Thus, being hospitalized and missing school has consequences to children’s and adolescents’ educational path, e.g., [1,35]. For example, literature has mentioned that hospitalized youngsters are more likely to experience learning difficulties [30], which may compromise their academic motivation [36] and entail emotional struggles (e.g., higher risk of psychosocial problems) [37]. Additionally, hospital stays may increase the risk of students disengaging from school, e.g., [2], mostly due to absence from school [38]. This is particularly important, because school disengagement may lead to early school dropout or educational underachievement [23]; academic failure, e.g., [1,39,40]; a lower likelihood of completing compulsory education [41]; or entering university [42]. Lastly, hospitalization may occur during critical transition points, such as starting school or key stages of learning and development, such as the onset of adolescence [2].

### 1.3. Hospitals as Learning Places

Hospitals are firstly a health space, but they can also be an educational place [43]. Specifically, pediatric hospitals may play an important role in the developmental and educational wellbeing of their inpatients by creating inpatient-centered environments embedded with educational opportunities [8,44]. Researchers, stakeholders, and hospital staff recognize the need to provide youth with opportunities to feel that, despite their school absence, they can still learn and practice school subject contents [2,45], thus reducing their isolation [46]. In fact, research identified the need to develop educational programs to mitigate the negative impact of school absence due to hospital stays. These programs should contribute to the maintenance of the relationships of the inpatient with their peers, e.g., [47], and promote students’ motivation [48,49,50], self-regulation learning (SRL) strategies, and school engagement [8,51]. Moreover, as the literature shows, to minimize the emerging difficulties during school re-entry, children should keep connected with academic and social activities, e.g., [52,53]. This highlights the significant role that engagement with education plays in this process [54]. Thus, by offering educational services, pediatric hospitals promote a continued link with learning [2,43], and maintain some kind of normalcy for their inpatients [55].

### 1.4. Adolescents and Technology

Information and communication technologies (ICT) have become an everyday tool among the current generation of children and adolescents when approaching education and learning, socialization, and leisure, both in the school and home contexts [56]. Therefore, the potential of these technologies has been explored in traditional and non-traditional educational settings [2].

In light of the educational needs of hospitalized youth and the importance of ICT among this population, research has been examining the use of technologies to keep youth connected to the school, social, and academic activities [46] through alternative approaches to learning [2,57]. In fact, off-the-shelf technologies are already being used [7] by youths in hospitals [2] and at home while recovering [58]. These technologies may help maintain engagement with the established educational path [59], ease learning, and promote well-being [47,57]. A literature review by Maor and Mitchem [7] confirmed that using technologies to withstand for the hospitalized youth’s academic path, as well as to impact on their well-being, are helpful (e.g., the “PEEBLES” program [60]). In this line of thought, the authors reinforce their claim that the use of technology in the hospital context could be an important tool to engage adolescents in learning [61]. Most of these studies used technology to maintain the connection between the hospitalized youngsters and their learning context [49,50,53,62], promoting their motivation to learn and facilitating their return to school. Most importantly, these ICT-based interventions help children engage in learning while in a nontraditional educational setting [49,63,64].

Despite the usefulness of these technologies, researchers warn of the existence of challenges or barriers when using technologies to support the learning needs of hospitalized youth, e.g., [65,66,67]. Specifically, pediatric patients highlight the following challenges: internet accessibility [50,61,64], technology-based struggles (e.g., maintenance), and lack of privacy [57]. When technologies may be used to facilitate the connection between the hospitalized youngster and the school, teachers also show a reluctance to use them [67]. Moreover, parents have concerns particularly regarding online safety [7] and exposure of the hospitalized child to their classmates [68]. Thus, some researchers alert to the importance of other context-related variables that should be considered when designing interventions. For example, the vulnerabilities and unpredictability of the hospital context, challenges related with the hospital settings, and the handling of health issues (e.g., hospital routine procedures) [7,55,62]. So, designing and using ICT in the pediatric ward must consider the idiosyncrasies of this specific setting [62].

However, there is still a gap in research regarding how technology can be used to meet the educational and well-being needs of hospitalized youngsters [2]. A systematic review on game technologies for pediatric patients reported that only 16% of the studies using game technology in the hospital context had education as a purpose (i.e., offer information about hospital procedures, educational courses, or a way of connecting with the patient’s classroom) [69]. In fact, most of these technologies target specific conditions either for children undergoing specific medical procedures (e.g., venipuncture, a potentially fearful treatment) or for children suffering from a specific pathology (e.g., cancer) [69]. Lastly, despite the existing studies that use technology as a learning tool to support children, there is a lack of studies targeting hospitalized youngsters’ school engagement and academic skills promotion.

### 1.5. The Present Study

Hospitalization is likely to have a negative impact on children’s and adolescents’ lives [35,70]. These negative consequences may be amplified for adolescents. Adolescence is commonly known as a critical time in an individual’s life [71,72] comprising significant physical, psychological, emotional, and cognitive changes [73,74]. Such changes imply achieving key developmental tasks that pose challenges and are favorable to vulnerabilities and risks [72,75], one of which is the youth’s risk of decreasing their school engagement [71,76,77] or becoming disengaged from school [71,78]. By itself, when hospitalized, disengagement with education due to hospitalization [63] adds to the equation. This is a concerning scenario since disengaged students are likely to experience academic distress [79]. Normalizing these adolescent’s lives [45] and ensuring they remain engaged in learning opportunities could be a crucial factor in meeting their needs.

School-aged children’s educational needs while hospitalized include the difficult connection with school, the need for academic support, low school engagement, and lack of self-regulation strategies [80,81]. Taking into consideration that (i) the average length of hospital stays in Portugal is small (4 to 5 days) [32], and (ii) that, in some situations, there is an insufficiency in tackling the difficulties evidenced by the hospitalized youth regarding study methods needed to cope with school demands, the present study was designed to overcome this difficulty by extending the intervention on SRL after hospital discharge. This type of accompaniment is especially pertinent when patients receive discharge but must stay at home for a period of time for a full recovery.

Therefore, the current investigation delivered an intervention program in a blended learning format [82,83]. Blended learning integrates face-to-face and online instructional activities aiming to improve and support learning [84]. Despite some challenges presented by this format [85], there is a continued interest in these environments since they offer opportunities for optimizing learning [86]. Besides, Lack’s [87] and Wu’s [88] reviews on performance outcomes concluded that there is little difference between students who enroll in online or blended courses and those who take face-to-face courses. Anchored on the premise that learning is a continuous process, blended learning should have synchronous and asynchronous moments [83] in which the SRL competencies play a crucial role, e.g., [8,51,89].

All considered, the present study aimed to equip pediatric patients with SRL skills, through a blended format. This training aimed to assist adolescents deal with hospitalization challenges, health issues, recovery, and re-entry into school, and, thus, promote their engagement with school tasks. Our study addressed three main aspects: (a) the negative impact of hospitalization on adolescents and its influence on engagement with school; (b) the expectation that every setting may be transformed in a learning space; and (c) the expectation that a blended format can be used to deliver programs to hospitalized adolescents with short hospital stays.

## 2. Materials and Methods

### 2.1. Context of the Study

The inpatient unit where the study was developed is a specialized ward for diagnosis, treatment, and monitoring of youngsters’ health conditions. This is a small unit with 30 beds, distributed in two aisles: one for young children (0 to 5 years old) and another for older children and adolescents (6 to 17 years old). The length of the stays varies mainly according to patient’s age and health condition, since this unit is not designed for youngsters with chronic illnesses or extreme health conditions. Younger children (0 to 5) stay on average 5 days (SD = 4.63; MIN = 1, MAX = 53), and older children and adolescents (6 to 17) stay on average 4 days (SD = 4.17; MIN = 1, MAX = 58).

### 2.2. Participants

Hospitalized adolescents attending the 7th, 8th, and 9th grades were invited to enroll in the present study. Two main reasons guided the option of recruiting inpatient youngsters from these grades: (i) compared to younger fellows, adolescents’ level of autonomy in using technology on their own is higher, this being a requirement by online learning environments [90]; and (ii) the learning (e.g., study time management, internal, and external distractors) and developmental demands (e.g., deep adolescence changes and peer group pressure) adolescents face during these school transition years may pose potential risks to overcome.

Fifty-seven youngsters were invited to participate and 20 accepted to enroll (attrition rate 65%); still, only 13 initiated the program (attrition rate 35%), and 10 completed the program (completion rate 77%). The main reasons for not enrolling in the program were related to difficulties to meet ICT inclusion criteria, for example, a lack of internet at home, not having a computer/laptop, or not having one with a camera. Moreover, despite accepting to participate, seven youngsters withdrew from the program before the sessions started due to schedule incompatibility. Of the remaining 13 youngsters who initiated the sessions, three gave up completing the program, one after Session 1, another after Session 3, and the third after Session 6. These dropouts were either due to reasons related with schedule incompatibility (*n* = 1) or to the inadequacy of the activities to their personal characteristics (e.g., lack of identification with the activities) (*n* = 2). Participants’ ages ranged between 12 and 16 years with a mean age of 13.7 (SD = 1.25). Of the 10 youngsters who completed the program seven were male (70%).

### 2.3. Theoretical Framework

The current program followed a model rooted in the social cognitive framework, according to which SRL involves three cyclical and interdependent phases. This cyclical model [91,92] is the foundation of the PLEE (Planning, Execution, and Evaluation) model [89,93], characterized by its recursive nature. Two paths of logic organize the three phases: (i) this process begins with the Planning phase through Execution to Evaluation, and (ii) each phase is informed by the same cyclical logic, containing the three phases in themselves, thus reinforcing the self-regulation logic. The Planning phase precedes the performance of the task and refers to the moment when it is expected that students define their goals and select learning strategies to help them achieve their goals. In the Execution phase the pre-established plan is implemented and monitored. Lastly, the Evaluation phase involves the analysis of the achieved outcome accounting for the established goals. The resulting information is critical to feed and initiate a new cycle and the subsequent tasks.

Assuming an agent role in their learning process enables students to take responsibility and control over their educational path, especially when facing difficulties [80]. Training in SRL strategies provides students with the necessary skills to influence their own cognitive and behavioral functioning [94]. In fact, students who use self-regulation strategies control their cognition, motivation, learning environments, and behaviors through cognitive and metacognitive processes [95]. Furthermore, students trained in SRL strategies (e.g., goal setting) show high school engagement levels and high academic performance [51,96,97].

### 2.4. Description of the Program

The present program used a set of educational narratives extracted from Testas’ (Mis)adventures five-book collection [98,99,100,101,102]. This collection was designed to promote 5th through 9th graders’ strategic learning through intentional training of SRL strategies. This story-tool collection was developed to support the student’s learning and training of SRL strategies and is based on the conviction that SRL can be promoted through narratives and modelling [103]. Thus, chapters have embedded the SRL strategies and, through the reading and analysis of the chapters’ contents with the help of a tutor, students learn vicariously. Throughout the books, the main character Testas describes how he handles health and school challenges (e.g., time management, memorizing, establishing goals, and asthma) and learning contents, how he helps his schoolmates, and describes his own process of exploring and using self-regulation strategies. Therefore, Testas functions as a close behavioral model, although being at the same time distant enough to allow the readers to reflect upon the use of learning strategies and build their own SRL model.

For the purposes of this intervention, all five books from the Testas’ (Mis)adventures collection [98,99,100,101,102] were read by three researchers who selected the chapters and the activities most suitable for the intervention. The set of chapters and activities was carefully chosen to guarantee a logical sequence so that the theoretical framework could be fully addressed (see Table 1). Furthermore, the chosen activities pertained to academic, health, and daily life content to meet students’ learning needs.

### 2.5. Procedure

Before undertaking the research, the project was approved by the University of Minho Ethics Committee for Research in Social and Human Sciences (CEICSH) (CEICSH 032/2019). CEICSH considered that the project followed requirements for good practice in human research in accordance with the Declaration of Helsinki. Prior to data collection, participants and parents/caregivers gave their written consent. Codes were assigned to identify the participants to protect confidentiality of the data.

The intervention program was organized in eight weekly individual sessions of approximately 60 minutes each. The development of the blended learning program occurred first in the hospital (face-to-face modality) and proceeded later at home (online modality). Each youngster had, at least, one session while staying hospitalized in the ward and then, after hospital discharge, the program was completed online by using Skype.

Sessions were conducted by two researchers trained on SRL who followed the established protocol. The program sessions were organized in a three-step sequence: (i) discussion and reflection about the chapter (which was delivered to the student before the session took place, except for the first session); (ii) solving practical tasks; and (iii) final reflection on the addressed contents and their application to the student’s personal and academic life. During the weekly sessions, the chapters were usually read beforehand by the participants, and the activities provided the opportunity for the youngsters to acquire, practice, and reflect upon their own learning process and the use of SRL strategies.

Repeated measures assessment was carried out in sessions one, four, and eight. Between sessions, a text message was sent to the youngster reminding him/her of the next session by posing a small challenge (e.g., “Hi, how is the reading of the text going? Try to underline the most important ideas (Testas’s thought: *Underline? Why so much work? Ok, ok, if I’m told to do so, that’s because it must be anyhow useful*…)”). Even though both researchers followed the established protocol, the practical examples provided were tailored to each student and, whenever necessary, some topics were further and deeper approached.

### 2.6. Implementation Fidelity

After each session, both researchers completed a report on adherence to the established protocol. A weekly briefing was set in order for researchers to describe the session steps followed, discuss constraints and difficulties faced, and talk about the impact of the activities on students’ development. These weekly meetings were supervised by the program coordinators to ensure that the guidelines were followed and guarantee that the program application was as similar as possible. Analysis of the fidelity reports and meetings revealed an agreement of 95%.

### 2.7. Instruments and Measures

#### 2.7.1. SRL Strategies Inventory

SRL strategies inventory [89,96] assesses the use of SRL strategies through nine items pertaining to the three PLEE phases: Planning (e.g., I plan before I begin writing. I think about what I want to say and how I need to write it); Execution (e.g., I select a calm place where I can be concentrated to study); and Evaluation (e.g., When I receive a grade, I think of what I can do to improve). Each item is scored on a five-point Likert scale ranging from one (never) to five (always). Cronbach’s alpha of the scale was 0.84.

#### 2.7.2. Self-Efficacy for SRL

Students’ belief in their capabilities to regulate their own learning through a variety of learning strategies was assessed by means of a 10-item questionnaire [89,103]. Each item begins with the phrase “How well can you…” completed with statements such as “…use strategies to comprehensively memorize the study material” or “…establish school goals for each subject and a plan to achieve those goals”. These items were responded according to a five-point Likert scale ranging from one (not very well) to five (very well). Cronbach alpha of the scale was 0.89.

#### 2.7.3. Perceived Instrumentality of the SRL Strategies

This questionnaire access instrumentality or perceived utility of the SRL strategies in the academic context through 10 items. Each item begins with the phrase “How useful you think it is to you to…” completed with the same statements as the Self-efficacy for SRL questionnaire. These items were responded according to a five-point Likert scale ranging from one (not very useful) to five (very useful) [89]. Cronbach’s alpha of the scale was 0.81.

### 2.8. Data Analysis

Table 1 provides the descriptive statistics for self-regulated learning, self-efficacy, and instrumentality across time. In general, findings from the Shapiro–Wilk test (fit for small samples [104,105]) indicate that all variables were reasonably normally distributed; in fact, the examination of the skewness and kurtosis statistics indicated that all values were within the range of ±2 [106]. For this reason, the parametric statistic was used. A Pearson correlation was used to analyze the relationships between the quantitative variables, while a Student’s t-test for related samples was used, for example, to examine the maturation effect. The analysis of the effect of the intervention in distinct subgroups comprised by the variables age, school year, grade retention, and engagement in intervention was conducted by analyzing the interaction (time (pre and post measures) x group) with a partially repeated measures ANOVA (DMPR) using gain scores (GS). DMPR allows to evaluate which group changes significantly over time and the GS analysis informs whether groups are distinct regarding time [107].

Following the What Works Clearinghouse [108] recommendations, the magnitude of the findings was evaluated using the value of the statistical significance (*p* value), the *d* of Cohen, and the improvement index. The improvement index (% Nov) indicates the percentage of non-overlapping of data in the groups in comparison or of the measures being compared within the same group [91]. Following Cohen [109], a “small” association is defined as ηp2 = 0.010 (equivalent to Cohen’s *d* = 0.20), a “medium” association is ηp2 = 0.059 (equivalent to Cohen’s *d* = 0.50), and a “large” association is ηp2 = 0.138 (equivalent to Cohen’s *d* = 0.80). The % Nov as follows: <7% insignificant distance; approximately 33% = medium; 57.4% = large; and >51% = very large. Finally, correlations equal or higher than 0.70 were considered relevant [110,111]. The analyses were run using SPSS V25 (IBM, Armonk, NY, USA).

## 3. Results

Results of the current study are presented in five sections. In the first three, relevant information is provided to assess whether there are enough guarantee conditions for the analysis of our main hypotheses, while in the last two the effect of the intervention is examined. Specifically, information is provided regarding the sample, the experimental reactivity, and the regression to the mean, as well as to the presence, or not, of experimental maturation. After checking the above, findings on the effectiveness of the intervention are presented (Section 4). Finally, as complementary analyzes, data on the effect of the intervention on SRL, SE, and INST depending on other variables, such as age, grade level, grade retention, and engagement in intervention, was examined (Section 5).

The corresponding data are presented in three Tables (i.e., Table 2, Table 3 and Table 4). Table 2 provides the descriptive statistics for the variables self-regulated learning (SRL), self-efficacy (SE), and perceived instrumentality (INST) in the three moments, and for each of the subgroups formed according to the variables age, grade level, grade retention, and engagement in intervention. The evaluation of the equivalence in the pretest measures has been established based on the criterion recommended by What Works Clearinghouse [112].

### 3.1. Description of the Profile of the Sample and Exam of the Relationships between the Variables Taken

Participants were students struggling to learn and succeed in school. For example, out of the 10 participants, half had repeated a school year. Acknowledging this educational scenario, we grouped the current sample according to age (A1 = 12–13 years old (*n* = 4); A2 = 14–16 years old (*n* = 6)), grade level (GL1 = 7 (*n* = 6); GL2 = 8–10 (*n* = 4)) and grade retention (NGR = students with no grade retention (*n* = 5); GR = students with grade retention (*n* = 5)). The four students in A1 were enrolled in the 7th grade and half had repeated one school grade. The six students in A2 were engaged in different grade levels (7–10), and half had repeated a school year, two of them twice. Moreover, age and grade level showed a moderate and negative correlation with SRL1 and (*r* = −0.568; *p* = 0.087 and *r* = −0.423; *p* = 0.224, respectively) and SE1 (*r* = −0.612; *p* = 0.060 and *r* = −0.692; *p* = 0.026, respectively), but not with the same variables in the following moments (e.g., SRL2 and SRL3) nor with any measure of the variable INST (see Table 2).

Table 3 presents descriptive statistics for the variables SRL, SE, and INST for the three moments; correlation of the age and grade level variables with SRL, SE, and INST in the three temporal moments; correlation of SRL, SE, and INST in the first and last moments; the Student t-test for related samples (Mid-Pre); and the Student t-test for related samples (mean of the pre and mid measurements and post-intervention measure: Post-m2Pre).

Table 4 presents the results of the partially repeated measures ANOVA and the analysis with change scores (variables between groups: age, grade level, grade retention, and engagement in intervention).

### 3.2. Evaluation of the Experimental Reactivity and Regression To Mean

Table 2 shows that standard deviations of SRL1 and SE1 are much higher than those of the SRL2 and SE2. Moreover, we found low correlations between both measures in the first and second measures (i.e., *pre* and *mid*), see Table 4. Analyzing both findings we concluded a strong reactivity in the first measure (i.e., *pre*), but not in the second measure (i.e., *mid*); that is there is no evidence that participants are altering their performance because of the attention that the study focuses on them. Furthermore, regression to the mean is not occurring because variance is not stable in the measures pre and mid. Regarding the variable INST, despite the stability of the variance, we found a strong correlation between the measures pre and mid which prevents regression to the mean. We can conclude no experimental reactivity for this variable.

### 3.3. Evaluation of Experimental Maturation

Irrespective of the variables, no statistical differences for the measures *pre* and *mid* were found (see Table 3). Moreover, we found a low effect size for SE (*d* = 0.20) and an even lower for SRL and INST (*d* = 0.15 and *d* = 0.12, respectively). Analyzing these findings together with those of the correlations between the three variables in the measures pre and mid (i.e., strong relationship between SRL and SE, but not with INST) we did not find an experimental maturation effect regarding the variable INST. This means that we found evidence that participants are not improving as a function of the passage of time independently of treatment. Regarding SRL and SE, the low correlation between the pre and mid measures, and the high standard error mean (${\bar{X}}_{S}=$ 0.199 and ${\bar{X}}_{S}=$ 0.202, respectively) when compared with INST (${\bar{X}}_{S}=$ 0.096) may indicate that, within this time frame, participants were behaving distinctly. This result may be due, for example, to participants’ grade retention distinct experiences.

### 3.4. Evaluation of the Efficacy of the Intervention

We found statistically significant differences for SRL, SE, and INST between the pre, mid, and post measures (SRL: *t* = 5.302, *p* < 0.001; SE: *t* = 2.956, *p* < 0.05; INST: *t* = 3.271, *p* < 0.01; see Table 3), in favor of the posttest in the three variables. We found very high effect sizes for SRL and SE (*d* = 1.252 and *d* = 1.075), and high for INST (*d* = 0.726). Considering this result together with the correlation between the two pre measures and the post measure, we may conclude that the intervention was efficacious in promoting participants SRL and INST. Results were slightly higher for SRL, which may indicate the sensitivity of the measure in capturing the effect of the intervention. Regarding the variable SE, despite the high effect size, the ${\bar{X}}_{S}$ has not diminished when compared with the maturation test, and the correlation between the pre, mid measures and the post measure is practically irrelevant. Moreover, the strong correlation between both SE measures, pre and post, with INST and the low correlation between the same measures with SRL may be indicating that the intervention helped students became more SE, but in a complementary way to SRL.

### 3.5. Evaluation of the Efficacy of the Intervention Considering Age, Grade Level, Grade Retention, and Engagement in Intervention

Despite a general positive impact of the intervention, data stressed distinct ways participants may improve in their self-regulation processes. To further analyze these differences and deepen our knowledge on the distinct impact of the intervention, we conducted ancillary analysis to examine the engagement of participants in the intervention. The aim was to learn how the effects of the intervention on SRL, INST, and SE could be related to participants’ Age, Grade Level, Grade Retention, and Engagement in Intervention.

As previously explained, age, grade level, and grade retention were organized in two levels, and engagement in intervention in three levels. The variable engagement in intervention was built considering three aspects: attendance, punctuality, and homework completion. Participants were gathered in three groups as follows: strong engagement group (E3), including participants who attended all sessions, were punctual, and did all the homework assigned in the sessions (*n* = 5). Regular engagement group (E2), comprising students who, despite attending all sessions, were not always punctual, nor did all the homework (*n* = 4). Lastly, the low engagement group (E1), comprising students that attended all sessions but were never punctual and did homework occasionally (*n* = 3). Table 3 shows the results on the effect of the variables age, grade level, grade retention, and engagement in intervention on SRL, SE, and INST.

Data from the partially repeated measures ANOVA (see Table 4) show that age, grade level, grade retention, and engagement in intervention interact in a significant way with the effect of the intervention on the dependent variables (SRL, SE and INST). However, it is noteworthy that the independent variables (i.e., age, grade level, grade retention, and engagement in intervention) influence the three dependent variables differently. Specifically, the effect of the intervention on SRL is related to age (*F* = 16.10, *p* < 0.01, *d* = 2.54); the effect of the intervention on SE is related both with age (*F* = 5.25, *p* = 0.051, *d* = 1.49) and grade level (*F* = 4.90, *p* = 0.058, *d* = 1.40), although in this case the significance is marginal; and the effect of the intervention on INST is related with grade level (*F* = 7.46, *p* < 0.05, *d* = 1.73), grade retention (*F* = 5.20, *p* = 0.052, *d* = 1.44), and engagement in intervention (*F* = 4.41, *p* = 0.058, *d* = 1.90). These results are consistent with those from the partially repeated measures ANOVA (already commented) and those from the change scores (in Table 3 see a comparation of the effect sizes of the relationships).

Finally, we addressed the particular ways how the interaction of the covariates (age, grade level, grade retention, and engagement in intervention) are related with the effect of the intervention on SRL, SE, and INST (see effects in Table 4). The analysis of the variables SRL and SE indicates that the intervention was effective depending on the age of participants. For example, older students scored higher in both variables. Data showed that the measure of SRL is likely to capture differences in students with younger ages as well as in older students, while SE only captured differences in older students (see Table 4).

Regarding the variable grade level, data from SE (marginally statistically significant, see Table 3) and INST (*p* = 0.026) show that students in GL2 benefited more from the intervention than students in GL1; in fact, students in GL1 did not improve their SE and INST.

Data from the variable INST shows that the effect of the intervention is related to the engagement in intervention in the sessions. The more the students were engaged in the program, the more their perception of instrumentality. Moreover, the impact of the program on students displaying low engagement was not statistically significant.

Interestingly, data presented in Table 4 regarding the three dependent variables (SRL, SE, and INST) show that older students and students in higher grade levels, when compared with younger counterparts, are those who benefited more from the program. Besides, focusing on the variable INST data shows that students with no grade retentions are those who benefited more from the intervention. Finally, we highlight as important findings the high effect sizes and the high improvement index (see Table 4), even in results statistically marginally significant [108].

## 4. Discussion

The present study aimed to describe and evaluate a blended learning intervention on SRL for hospitalized school-aged adolescents. The program had two sequential formats: It was delivered firstly face-to-face on-site and, upon discharge, continued online at home. This way, the present program addressed the youth educational needs and considered the context particularities regarding and unpredictability already addressed. The worldwide recommendations [117] regarding development and implementation of programs for pediatric inpatients were also considered.

### 4.1. Main Results

Globally, the intervention was efficacious in promoting participants’ use of SRL strategies and in improving their perceived instrumentality of SRL strategies. Furthermore, results indicate that adolescents’ self-efficacy for SRL improved, but in a complementary way to SRL. These findings extend previous research that reports the benefits of participating in SRL training programs using story-tools, e.g., [51,89,96,118], and in hospital-based psychoeducational interventions [4,8]. Additionally, results are consistent with literature showing that adolescent SRL strategies can be enhanced with appropriate training [79,96] and methodologies (e.g., story-tools and SRL-based activities), even when there are a limited number of sessions [79].

Although the core goal of the intervention was the promotion of SRL competencies, results indicate that adolescents became more self-efficacious in a complementary way to SRL. In fact, the self-regulatory processes are influenced by motivational beliefs [92], self-efficacy being an important variable in SRL [119]. Specifically, the personal use of SRL strategies is related to one’s self-efficacy beliefs, i.e., there is a positive relationship between one’s perception of competence and the exercise of that competence [120,121,122,123]. Moreover, research shows that training in self-regulatory skills can increase students’ self-efficacy beliefs [123] and academic achievement [124]. Additionally, to this general approach to data, findings will be further discussed in relation to the learning context and the differential impact of the program.

### 4.2. Hospital Context

Literature suggests that SRL is context dependent [125], proposing that the enactment on SRL strategies by a student may be influenced by the singular characteristics of the learning environment. It is important to acknowledge that participants improved their use of and their perceived instrumentality of SRL strategies, engaged in the program while hospitalized, later at home when recovering, and/or subsequently when re-entering school.

Findings of the present study are consistent with previous research that used tailored programs as a mean to address the educational needs of hospitalized youth, e.g., [8,10,126]. Typically, current educational interventions are implemented in the context of Hospital Schools for inpatients with long or repeated admissions [127]. The program implemented acknowledges the need to address the educational challenges faced by adolescents who have a short hospital stay and are discharged to recover at home and/or return to school. Following a preventive approach [8] and the SRL literature recommendations, e.g., [128], the program aimed at SRL competence promotion, highlighting adolescents’ proactive role in an ecological environment. Additionally, these SRL competences enable youths to face situations that are crucial for their engagement in education.

### 4.3. Format of Delivery: Face-To-Face, Online, and Blended

In the field of clinical psychology, several authors have been researching online therapy and comparing it to face-to-face therapy. A systematic review and meta-analysis by Carlbring and colleagues [129] show that both formats are equally effective. Cognitive behavioral therapy delivered online also has been considered a promising way to increase access to the treatment [130], particularly among young patients [131]. Overall, there is a voluminous work on internet-based interventions that support its use for delivering psychotherapy for mental disorders [132].

In recent years, educational psychology has also been exploring the field of online interventions. Cerezo and colleagues [133], and Núñez and colleagues [134], used a virtual format to deliver a training program in SRL strategies for college students with positive impact on their academic success. Even though the participants were from a different educational and developmental level, this corpus of findings together with the present study are promising in that ICT is a viable platform to deliver training in SRL strategies.

To the best of our knowledge there is limited research on blended educational interventions bridging the hospital to home while adolescents are still recovering their health. Findings of the present study add to Spanjers and colleagues’ [86] research reinforcing that the blended format offers opportunities to improve learning.

### 4.4. Differential Impact of the Program

Despite a general positive impact of the intervention, data stressed distinct ways participants may improve in their self-regulatory processes. Findings showed a differential impact of the program as a function of the age, grade level, grade retention, and engagement in intervention. Analyzing these differences is, therefore, crucial to shed light on how hospitalized or in recovery adolescents self-regulate their learning, perceive SRL strategies usefulness to achieve their goals, and report themselves as self-efficacious in using them.

#### 4.4.1. Age

The results of the present study indicate that older adolescents showed less SRL competencies and self-efficacy at the beginning of the intervention and were the ones who had better scores on both variables at the end of the program. Hence, older adolescents were the ones who benefited the most with the intervention comparing to their younger counterparts. These results are not consistent with those of previous research. A meta-analysis on self-regulation training programs indicates that younger students benefit more from interventions designed to promote study skill competences [129]. Research also shows that it is easier to teach SRL strategies to younger students as they are on the onset of building their own set of learning strategies and self-efficacy perceptions [79,135]. In fact, SRL strategies are amenable to be taught [48,136], but they require that the student assume an agent role, which is crucial for the development of the SRL process. Personal agency develops throughout life, but it is on the onset of adolescence that students begin to develop agency as they are expected to assume responsibility for their learning process at this stage [137]. Moreover, a robust sense of personal agency is anchored in self-efficacy beliefs in one’s self-regulative skill to achieve goals [137]. As current data indicates, younger participants show lower SRL and self-efficacy than their counterparts, which may be indicating that they are in the process of developing an agent role. Younger adolescents also might have been more concerned with their hospitalization [9], whereas older ones, when faced with absence from school and the fear of falling behind, may have been more open to broaden their strategy repertoire. Moreover, it is important to reflect upon the fact that the intervention was, in part, delivered through technology. Older participants may have digital literacy further developed, thus being more ready to learn through a digital means. Digital literacy implies the use of digital tools to communicate effectively with others and create meaning [138], for which metacognitive and self-regulated skills are needed [139].

#### 4.4.2. Grade Level

Adolescents from higher grade levels, when compared to their counterparts in the 7th grade, showed better scores on self-efficacy and instrumentality whereas those in the 7th grade did not improve on these variables. Participants were likely to perceive their schoolwork as an important way to achieve success as they progressed from the 7th to 10th grade. Current data, in contrast to prior studies, which found that self-efficacy for SRL decreases as youths progress through school, e.g., [140], are consistent with recent findings by Shin and colleagues [141] who found that overall students’ perceived instrumentality increased over time. Self-efficacy and instrumentality influence the way students approach their learning process. Specifically, intentional training on learning strategies is closely related to students’ self-efficacy beliefs [140] and to its perceived utility to improve the learning process. Instrumentality of SRL strategies and the perceived demands of the learning context play an important role on the learning process [89] and may help explain students’ learning process. In fact, adolescents involuntary distancing from school, due to hospitalization, presented additional challenges to the intervention that may have spurred the usefulness of the SRL strategies.

#### 4.4.3. Grade Retention

Results indicate that students with no grade retentions improved more on their instrumentality when compared to the students that failed at least one academic year. This result poses an educational challenge since students with grade retentions do not seem to be fully grasping the utility of using SRL strategies as a mean to deal with academic challenges (e.g., time management and note taking). Instrumentality is key because students with strong perceptions of instrumentality are aware of the importance of present tasks for future goals, e.g., [142].

The interruption on their normal lives due to hospitalization added new challenges to their academic path; note that these adolescents were students with a record of grade retention, which might have hindered their availability to understand the instrumentality of the SRL strategies they were learning. The SRL contents discussed in the program might also not have met their expectations and immediate academic challenges, possibly focused on recovering their health. Overall, these results call for a careful analysis and alert for the importance of considering the specificities of the participants’ academic background in future studies.

#### 4.4.4. Engagement in Intervention

Interestingly, data show that students improved their perception of the instrumentality of SRL strategies when they were more engaged in the program. Perceiving the usefulness of the SRL strategies for the learning process is a critical motivational variable for the student’s engagement in learning [79,143]. Engagement pertains to the involvement in an activity which entails personal investment and instrumentality pertains to the perception of usefulness. Both may be promoted through the opportunity to experience success while participating in the program [51,96]. Engaging in the program and having the opportunity to learn the nature of the SRL strategies, how, and when to use them effectively, may have also provided adolescents with the opportunity to practice these strategies allowing to perceive their utility. When adolescents understand the instrumentality of using SRL strategies to achieve their self-set goals, they are more likely to use them, e.g., [119], and to achieve success.

## 5. Limitations, Future Research, and Educational Implications

The preliminary results of this study should be interpreted with caution considering the limitations and challenges faced along the implementation. Despite the difficulties to enroll hospitalized adolescents, future research could consider gathering a larger sample, using a control group and investigate the long-term effects of this intervention. Future studies could also evaluate the impact of the program in school re-entry; this is an important developmental challenge for hospitalized adolescents that merits further research.

Unlike previous research, which highlights the importance of intervening as early as possible [96,118,135], this study showed that older adolescents improved more than their counterparts. Future research could investigate distinct contextual factors (e.g., ICT platform, hospitalization, home recovery, and blended learning) of the program implementation that may help explain these findings. Literature on the efficacy of SRL training programs is vast, with different populations (e.g., gypsy community), ages (e.g., primary and college students), and formats (e.g., mentoring), e.g., [96]. This study approaches ICT, in a blended format, as a viable mean to deliver interventions focused on training SRL with story-tools. Therefore, in future studies it could be important, besides having a control group (a limitation of this study), to include another two experimental groups: one doing the training program in an online format and another in a face-to-face format. Thus, a comparison of the efficacy of the same SRL training program delivered online, face-to-face, and blended could be analyzed and help enlighten if ICT is a valid educational resource for the training of SRL strategies. Moreover, reflecting on the differential impact of the intervention on SRL in older students, future research could investigate the relation between digital literacy and SRL skills. Acknowledging that instrumentality is key to gear agency, program interventions could focus on this variable as a mean to deal with students displaying low engagement and with grade retentions.

The present results add to the corpus of knowledge by indicating that hospitals may be considered suited educational contexts [43] to deliver interventions focused on the training of SRL strategies [8,72]. Further research is needed to support current preliminary findings, but data indicated that blended formats may be relevant tools to make a bridge from hospital to home/school and address adolescents’ educational needs. Equipping youth with SRL strategies repertoire enables them to take responsibility and assume an agent role in their learning process. The ultimate goal of this intervention was to provide students with strategies and an SRL framework to help them overcome obstacles that may challenge their goal attainment and overall health and well-being.

## 6. Conclusions

Despite the challenges involved in conducting research in ecological settings, and the difficulties for testing hypotheses with small samples with high variability, we learned that the current intervention was effective. The strict control of the research design, the absence of threats to internal validity, and the strength of the effect size may help explain these findings. Overall, regardless of the limitations of the study, and in accordance with previous research, our findings alert for the potential of using story-tools to train SRL strategies despite participants’ characteristics, context, and delivery format. Finally, this study suggests the need to consider the potential of blended learning to deliver educational training making a bridge from hospital to home.

## Figures and Tables

**Table 1:** Content and self-regulation learning (SRL) strategies organization throughout the programs’ sessions.

Sessions	SRL Strategies and Contents Addressed	Examples of Activities
Session 1 *Hi! I’m Testas*	- Presentation and definition of session rules - Reflection about the learning process and the youngster’s role as a student	- Questions about how the youngster perceives himself/herself as a student
Session 2 *Testas’ efforts to explain the PLEE model*	- Initial exploration of the cyclical PLEE model - Transference of the PLEE model to the health context - Planning and management of study time	- Organize *Testas’* explanation of an asthma management situation according to the PLEE phases - To do list - Timetable organization
Session 3 *Planning with Testas*	- Establishing goals (specific, realistic, and assessable) - Long- and short-term goals - Exploration of internal and external distractors	- Establish goals - The Oscars of distractors
Session 4 *I’m Execution, the worker/the working one*	- Reflection about difficulties and obstacles through varied contexts - Definition of strategies to control internal and external distractors and improve attention	- What are my difficulties? - The distracted manual
Session 5 *Let’s start executing*	- Introduction to study strategies such as information organization (e.g., summaries), note taking strategies, study strategies (e.g., memorization techniques)	- Notebooks are important - What I should or shouldn’t do when taking notes
Session 6 *Test anxiety: remedy is demanded*	- Notebook organization - Preparation strategies for tests - Test anxiety and strategies	- Consolidation activity: Identify the strategies used by each student - Acute test anxiety
Session 7 *Evaluation: D. Antonieta’s party gone wrong*	- Plan evaluation regarding different contexts (e.g., personal life, school) - PLEE phases consolidation activity	- Which error did D. Antonieta commit? - Errors as opportunities to re-plan - Consolidation activity: Identify each phase in popular sayings
Session 8 *It’s time to say goodbye*	- PLEE phases consolidation activity - Good-bye activity	- PLEE phases in Pancho’s story - It´s time to say goodbye. What do you want to say to *Testas*

**Table 2:** Descriptive statistics of the variables SRL, self-efficacy (SE), and perceived instrumentality (INST) for the whole sample in the three measurement occasions, and for each one of the subgroups formed according to the variables age, grade level, grade retention and engagement in intervention.

	Pre	Mid	Post	Mid-Pre	MD_g_		m2Pre	Post-mMid
					Pre	Mid		
**SRL**								
TS	3.83 (0.70)	3.93 (0.25)	4.4 (0.43)	0.09 (0.63)			3.87 (0.42)	0.53 (0.32)
A1	4.19 (0.28)	3.84 (0.24)	4.25 (0.40)	−0.35 (0.18)	0.60	0.13	4.01 (0.24)	0.23 (0.26)
A2	3.59 (0.82)	3.98 (0.27)	4.52 (0.44)	0.38 (0.65)			3.78 (0.51)	0.73 (0.14)
GL1	3.93 (0.76)	3.83 (0.27)	4.28 (0.47)	−0.09 (0.58)	0.23	0.22	3.87 (0.48)	0.40 (0.33)
GL2	3.69 (0.69)	4.06 (0.16)	4.61 (0.29)	0.36 (0.67)			3.87 (0.37)	0.73 (0.18)
NGR	4.04 (0.53)	3.93 (0.27)	4.38 (0.49)	−0.11 (0.30)	0.42	0	3.98 (0.33)	0.39 (0.25)
GR	3.62 (0.85)	3.93 (0.26)	4.44 (0.41)	0.30 (0.72)			3.77 (0.51)	0.67 (0.24)
ENG1	3.19 (0.85)	3.88 (0.33)	4.30 (0.45)	0.68 (0.69)			3.53 (0.54)	0.77 (0.25)
ENG2	4.08 (0.59)	3.97 (0.12)	4.47 (0.29)	−0.11 (0.53)	0.96 ^	0.04	4.02 (0.33)	0.45 (0.29)
ENG3	4.15 (0.28) ^A^	3.92 (0.38)	4.44 (0.68)	−0.23 (0.32			4.03 (0.29)	0.41 (0.38)
**SE**								
TS	3.92 (0.69)	4.04 (0.44)	4.44 (0.36)	0.12 (0.63)			3.98 (0.48)	0.46 (0.49)
A1	4.35 (0.64)	4.05 (0.66)	4.30 (0.41)	−0.30 (0.24)	0.71	0.012	4.20 (0.63)	0.10 (0.32)
A2	3.63 (0.60)	4.03 (0.30)	4.53 (0.32)	0.40 (0.26)			3.83 (0.33)	0.70 (0.45)
GL1	4.17 (0.62)	4.02 (0.51)	4.32 (0.35)	−0.15 (0.37)	0.61	0.05	4.09 (0.53)	0.22 (0.44)
GL2	3.55 (0.69)	4.08 (0.38)	4.63 (0.31)	0.52 (0.79)			3.81 (0.38)	0.81 (0.36)
NGR	3.86 (0.44)	3.84 (0.43)	4.40 (0.42)	−0.02 (0.52)	0.12	0.40	3.85 (0.34)	0.55 (0.36)
GR	3.98 (0.93)	4.24 (0.39)	4.48 (0.33)	0.26 (0.77)			4.11 (0.59)	0.37 (0.63)
ENG1	3.70 (1.18)	4.33 (0.51)	4.70 (0.17)	0.63 (0.80)			4.01 (0.81)	0.68 (0.64)
ENG2	4.10 (0.56)	3.98 (0.21)	4.15 (0.06)	−0.12 (0.57)	0.40	0.35	4.03 (0.30)	0.11 (0.32)
ENG3	3.90 (0.36) ^A^	3.83 (0.59)	4.57 (0.49)	−0.06 (0.35)			3.86 (0.45)	0.70 (0.35)
**INST**								
TS	4.17 (0.52)	4.23 (0.45)	4.53 (0.44)	0.06 (0.46)			4.2 (0.46)	0.33 (0.32)
A1	4.15 (0.66) ^A^	4.32 (0.68)	4.43 (0.54)	0.17 (0.28)	0.33	0.15	4.23 (0.65)	0.19 (0.14)
A2	4.18 (0.48)	4.17 (0.27)	4.60 (0.40)	−0.01 (0.31)			4.17 (0.35)	0.42 (0.38)
GL1	4.25 (0.56) ^A^	4.27 (0.56)	4.42 (0.43)	0.01 (0.33)	0.20	0.09	4.25 (0.53)	0.16 (0.25)
GL2	4.05 (0.52)	4.18 (0.29)	4.70 (0.48)	0.12 (0.28)			4.11 (0.39)	0.59 (0.23)
NGR	3.82 (0.44) ^A^	4.06 (0.48)	4.46 (0.59)	0.24 (0.30)	0.70 ^*^1^	0.34	3.94 (0.43)	0.52 (0.25)
GR	4.52 (0.34) ^A^	4.40 (0.39)	4.60 (0.29)	−0.12 (0.17)			4.46 (0.35)	0.14 (0.28)
ENG1	4.73 (0.25) ^A^	4.60 (0.35)	4.70 (0.36)	−0.13 (0.15)			4.66 (0.29)	0.03 (0.30)
ENG2	3.93 (0.43)	4.15 (0.37)	4.38 (0.26)	0.22 (0.41)	0.80 ^*^1^	0.45	4.03 (0.34)	0.34 (0.14)
ENG3	3.93 (0.46) ^A^	3.97 (0.51)	4.57 (0.75)	0.03 (0.15)			3.95 (0.48)	0.62 (0.28)

Note: TS = total sample; A = age. A1 and A2, 12–13 years old (*n* = 4) and 14–16 years old (*n* = 6), respectively; GL = grade level. GL1 and GL2, 7th grade (*n* = 6) and 8th–10th grades (*n* = 4), respectively; GR = grade retention. NGR and GR, no grade retention and grade retention, respectively (*n* = 5 in both); ENG = engagement in intervention. ENG1, ENG2 and ENG3, null (*n* = 3), high (*n* = 4) and very high (*n* = 3), respectively; ^A^ = standard deviation less or equal in the first measure Pre than in the second one; |MD_g_|= mean absolute differences between the groups defined by the variables: age, grade level, grade retention and engagement (in this case, between the two most extreme means) in the variables SRL, SE, and INST in the measurements Pre and Mid; ^ = if |MD_g_| > 0.05ED = if |MD_g_| is greater than 0.05 standard deviations in absolute value (based on the variation of that characteristic in the pooled sample). The values 0.05SD are (in Pre: SRL = 0.73; SE = 0.72; INST = 0.54) and (in Mid: SRL = 0.26; SE = 0.46; Inst = 0.47); * = statistically significant difference of means applying Benjamini–Hochberg correction for multiple outcome measures tested with multiple comparison groups like Table 3. In this case M = 24; ^1^ = (*p* = 0.023; *p*_B-H_ = 0.010); rest, see Table 2 and Table 3.

**Table 3:** Descriptive statistics, correlations, and results for testing the maturation and intervention hypothesis.

	**Descriptive statistics ^1^**									
	**Pre**	**Mid**	**Post**	**Mid-Pre**	**m2Pre**	**Post-m2Pre**				
SRL	3.83 (0.70)	3.93 (0.25)	4.4 (0.43)	0.09 (0.63)	3.87 (0.42)	0.53 (0.32)				
SE	3.92 (0.69)	4.04 (0.44)	4.44 (0.36)	0.12 (0.63)	3.98 (0.48)	0.46 (0.49)				
INST	4.17 (0.52)	4.23 (0.45)	4.53 (0.44)	0.06 (0.30)	4.2 (0.46)	0.33 (0.32)				
	**Correlation of the variables age and grade level with the variables SRL, SE and INST in the three measurement occasions**									
	**Grade Level**	**SRL1**	**SRL2**	**SRL3**	**SE1**	**SE2**	**SE3**	**INST1**	**INST2**	**INST3**
Age	0.846 **	−0.568 ^2^	0.362	0.327	−0.612 ^3^	0.246	0.354	0.138	−0.002	0.237
Grade L.	1	−0.423 ^4^	0.376	0.222	−0.692 *	0.100	0.241	−0.138	−0.072	0.163
	**Correlation of the variables SRL, SE and INST to each other on the first and last measurement occasion (left and right respectively)**									
	**SRL1**	**SE1**	**INST1**			**SRL3**	**SE3**	**INST3**		
SRL1	1	0.735 *	−0.109		SRL3	1	0.416	0.788 **		
SE1		1	0.326		SE3		1	0.783 **		
	**Paired Student’s *t*-test ^5^ for mid-pre. Test of the maturation hypothesis**									
	**R_Pre-Mid_**	${\bar{Y}}_{D}$	${\bar{X}}_{SD}$	***T***	***p***	**95% CI_D_ (ll)**	**95% CI_D_ (ul)**	***p*_B-H_**	***d*^6^**	**% N_ov_**
SRL	0.454	0.092	0.199	0.460	0.656	−0.359	0.543	--	0.151	7.7%
SE	0.427	0.120	0.202	0.594	0.567	−0.337	0.577	--	0.201	14.7%
INST	0.817	0.060	0.096	0.627	0.546	−0.156	0.276	--	0.12	7.7%
	**Paired Student’s *t*-test ^5^ for Post-m2Pre. Test of the effect of the intervention hypothesis**									
	**R_m2Pre-Post_**	${\bar{Y}}_{D}$	${\bar{X}}_{SD}$	***T***	***p***	**95% CI_D_ (ll)**	**95% CI_D_ (ul)**		***d*^6^**	**% N_ov_**
SRL	0.721	0.532	0.100	5.302	0.000	0.305	0.759	0.008	1.252	62.2%
SE	0.339	0.460	0.156	2.956	0.016	0.108	0.812	0.016	1.075	58.9%
INST	0.754	0.330	0.101	3.271	0.010	0.102	0.558	0.025	0.726	43%

Note: Block 1 (top of the Table): descriptive statistics of the variables SRL, SE, and INST in the whole sample in the three measurement occasions. Block 2: correlation of the variables age and grade level with the variables SRL, SE, and INST in the three measurement occasions. Block 3: correlation of the variables SRL, SE and INST to each other on the first and last measurement occasion (left and right respectively). Block 4 and Block 5: Statistics derived from the paired Student’s *t*-test for Mid-pre related samples, and for Post-m2Pre related samples respectively. Pre, Mid and Post = pre-treatment 1 and 2, and post-treatment measure; ^1^ = in Table cells mean (standard deviation); Mid-Pre = mean of the change scores between mid and Pre; m2Pre = mean of the two measures Pre; Post-m2Pre = mean of the change scores between Post and m2Pre; ^2, 3^, and ^4^ = *p* = 0.087, *p* = 0.060 and *p* = 0.224, respectively; y; * and ** = *p* < 0.05 and *p* < 0.01 (2-tailed); ^5^ = degree of freedom (*df*) = 9; R_Pre-mid y_ R_m2Pre-Post_ = Pearson correlation between Pre and Mid, and between m2Pre and the Post, respectively; ${\bar{Y}}_{D}=$ *difference* between *means*; ${\bar{X}}_{S}=$ pooled *standard deviation*; *t* = *t*-test value; *p* = *p* value; 95% CI_D_ = 95% confidence interval of the difference (ll-ul = lower and upper limits respectively); *p*_B-H_ = Benjamini–Hochberg correction for multiple outcome measures tested with a single comparison group, px’=xαM (where x is the rank for px, with *x* = 1, 2, …, m; m is the total number of tests, 6 in this case, and *α* is the target level of statistical significance, 0.05) [113]; ^6^ = effect size calculated by Cohen’s d corrected for paired *t*-test ([114]; see [115] (p. 228)); % N_ov_ = percent of non-overlap [109] (pp. 21–23) or improvement index [108] (p. 15).

**Table 4:** Results of the interaction effect in the mixed-design ANOVA, and results of the analysis of change scores between groups of variables: age, grade level, grade retention, and engagement in intervention.

	Mixed-Design ANOVA (Interaction Effect)		Change Scores ^B^	
	Summary Statistics of the Model ^A^	Simple Effects	Summary Statistics of the Model ^C^	MD
	**SRL**			
Age	*F* = 16.1; MSE = 0.019; *p* = 0.004; *d* ^1^ = 2.54; % N_ov_ > 90%; 1 − *β* = 0.937; *p*_B-H_ = 0.027	A1 = * Post-Pre ^D^ = 0.231; *p* = 0.045; *p*_B-H_ = 0.050 A2 = * Post-Pre = 0.733; *p* = 0.000; *p*_B-H_ = 0.056	MSE = 0.038; *d* = 2.58; % N_ov_ > 90%	* A2 − A1 = 0.502
	**SE**			
Age	*F* = 5.25; MSE = 0.082; *p* = 0.051; *d* ^2^ = 1.49; % N_ov_ = 73.1%; 1 − *β* = 0.522; *p*_B-H_ = 0.055	A1 = --- A2 = * Post-Pre = 0.700; *p* = 0.003; *p*_B-H_ = 0.022	MSE = 0.164; *d* = 1.478; % N_ov_ = 70.7%	* A2 − A1 = 0.600
Grade level	*F* = 4.90; MSE = 0.084; *p* = 0.058; *d* ^3^ = 1.40; % N_ov_ = 70.7%; 1 − *β* = 0.647; *p*_B-H_ = 0.066	GL1 = --- GL2 = * Post-Pre = 0.812; *p* = 0.004; *p*_B-H_ = 0.027	MSE = 0.169; *d* = 1.427; % N_ov_ = 68.1%	* GL2 − GL1 = 0.588
	**INST**			
Grade level	*F* = 7.46; MSE = 0.030; *p* = 0.026; *d* ^4^ = 1.73; % N_ov_ = 79.4%; 1 − *β* = 0.801; *p*_B-H_ = 0.044	GL1 = --- GL2 = * Post-Pre = 0.587; *p* = 0.001; *p*_B-H_ = 0.011	MSE = 0.059; *d* = 1.762; % N_ov_ = 75.4%	* GL2 − GL1 = 0.429
Grade retention	*F* = 5.20; MSE = 0.035; *p* = 0.052; *d* ^5^ = 1.44; % N_ov_ = 73.1%; 1 − *β* = 0.669; *p*_B-H_ = 0.061	GR = --- NGR = * Post-Pre = 0.520; *p* = 0.002; *p*_B-H_ = 0.016	MSE = 0.069; *d* = 1.442; % N_ov_ = 68.1%	* NGR − GR = 0.380
Engagement in intervention	*F* = 4.41; MSE = 0.029; *p* = 0.058; *d* ^6^ = 1.90; % N_ov_ = 81.1%; 1 − *β* = 0.719; *p*_B-H_ = 0.066	ENG1 = * Post-Pre = 0.033; *p* = 0.817; ENG2 = * Post-Pre = 0.338; *p* = 0.026; *p*_B-H_ = 0.044 ENG3 = * Post-Pre = 0.617; *p* = 0.003; *p*_B-H_ = 0.022	MSE = 0.058; *d* = 2.01; % N_ov_ > 81%	* ENG2 − ENG1 = 0.364 * ENG3 − ENG2 = 0.279 * ENG3 − ENG1 = 0.583

Note: A = age. A1 and A2, 12–13 years old (*n* = 4) and 14–16 years old (*n* = 6) respectively; GL= grade level. GL1 and GL2, 7 grade (*n* = 6) and 8–10 grades (*n* = 4), respectively; GR = grade retention. NGR and GR, no grade retention and grade retention, respectively (*n* = 5 in both); ENG = engagement in intervention. ENG1, ENG2 and ENG3, null (*n* = 3), high (*n* = 4), and very high (*n* = 3), respectively; *F*, MSE= Interaction effect, *F* value, and mean square error (MSE), respectively; ^A^ = the *df* are 1 and 8 in all the comparisons except in ENG which are 2 and 7 for the interaction effect and the *error term* (contrast term) respectively; *d* ^1–6^ = η^2^ has been transformed into Cohen’s *d* (according to [109]). The partial η^2^ values respectively are 0.667, 0.397, 0.380, 0.483, 0.394, 0.558; ^B^ = t = $\sqrt{F}$ of the Mixed-design ANOVA, in order from top to bottom in the table, 4.01, 2.29, 2.21, 2.73, 2.28 y 4.41 respectively. The *p*-value is also the same; ^C^ = the calculation Cohen’s *d* for change scores has been made according to [116] p. 119); ^D^ = in this table is m2Pre (see Table 2); 1 − *β* = the empirical power of the statistical test; * = indicates the highest mean; MD = mean differences; *p*_B-H_ = Benjamini–Hochberg correction for multiple outcome measures tested with multiple comparison groups, =(iM)Q (where *i* = the individual *p*-value’s rank; M = total number of tests -in this case, they are 15 for each variable. In total they are 45-, and Q = the false discovery rate (a percentage, chosen by you, the calculation for the critical value with a false discovery rate of 25%) [111]. Rest, see Table 2.
